# Integrating Clinical and Genetic Analysis of Perineural Invasion in Head and Neck Squamous Cell Carcinoma

**DOI:** 10.3389/fonc.2019.00434

**Published:** 2019-05-31

**Authors:** Ze Zhang, Ruoyan Liu, Rui Jin, Yanling Fan, Tingting Li, Yanjie Shuai, Xingchen Li, Xudong Wang, Jingtao Luo

**Affiliations:** ^1^Department of Maxillofacial and Otorhinolaryngology Oncology and Department of Head and Neck Oncology, Tianjin Medical University Cancer Institute and Hospital, National Clinical Research Center for Cancer, Tianjin, China; ^2^Key Laboratory of Cancer Prevention and Therapy, Tianjin Medical University Cancer Institute and Hospital, National Clinical Research Center for Cancer, Tianjin, China; ^3^Tianjin's Clinical Research Center for Cancer, Tianjin, China; ^4^Department of Gynecologic Oncology, Tianjin Medical University Cancer Institute and Hospital, National Clinical Research Center for Cancer, Tianjin, China; ^5^Public Laboratory, Tianjin Medical University Cancer Institute and Hospital, National Clinical Research Center for Cancer, Tianjin, China; ^6^Department of General Surgery, Nanfang Hospital, Southern Medical University, Guangzhou, China

**Keywords:** perineural invasion, HNSCC, WGCNA, TCGA, single-cell sequencing

## Abstract

**Introduction:** Perineural invasion (PNI), a key pathological feature of head and neck squamous cell carcinoma (HNSCC), predicts poor survival. However, the associated clinical characteristics remain uncertain, and the molecular mechanisms are largely unknown.

**Materials and methods:** HNSCC gene expression and corresponding clinical data were downloaded from The Cancer Genome Atlas (TCGA). Prognostic subgroup analysis was performed, and potential PNI risk factors were assessed with logistic regression. PNI-associated gene coexpression modules were identified with weighted gene coexpression network analysis (WGCNA), and key module gene functions and the roles of non-malignant cells in PNI were evaluated with a single-cell transcriptomic dataset (GSE103322).

**Results:** PNI was significantly inversely associated with overall survival (HR, 2.08; 95% CI, 1.27 to 3.40; *P* = 0.004), especially in advanced patients (HR, 2.62; 95% CI, 1.48 to 4.64; *P* < 0.001). Age, gender, smoking history, and alcohol history were not risk factors. HPV-positive cases were less likely than HPV-negative cases to develop PNI (OR, 0.28; 95% CI, 0.09 to 0.76; *P* = 0.017). WGCNA identified a unique significantly PNI-associated coexpression module containing 357 genes, with 12 hub genes (TIMP2, MIR198, LAMA4, FAM198B, MIR4649, COL5A1, COL1A2, OLFML2B, MMP2, FBN1, ADAM12, and PDGFRB). Single-cell transcriptomic data analysis revealed that the genes in the PNI-associated module correlated with the signatures “EMT,” “metastasis,” and “invasion.” Among non-malignant cells, fibroblasts had relatively high expression of the key genes.

**Conclusion:** At the molecular and omic levels, we verified that PNI in HNSCC is a process of invasion rather than simple diffusion. Fibroblasts probably play an important role in PNI.

**Novelty & Impact Statements**

The study is a thorough analysis of PNI in HNSCC from the clinical level to the molecular level and presents the first description of cancer-related PNI from the omics perspective to date as far as we know. We verified that PNI in HNSCC is a process of invasion rather than simple diffusion, at the molecular and omic levels. Fibroblasts were found to probably play an important role in PNI by analyzing single-cell transcriptomic data.

## Introduction

Head and neck cancer is one of the most common types of malignant tumor worldwide and accounts for nearly 5% of new cancer cases each year (1). Approximately 95% of head and neck cancer cases are diagnosed as head and neck squamous cell carcinoma (HNSCC). However, the survival rates of HNSCC remain relatively poor (2). Perineural invasion (PNI) has emerged as a key pathological feature of HNSCC and is a predictor of poor survival (3, 4).

According to the existing literature, the clinical characteristics associated with PNI remain uncertain. For example, the incidence of PNI in HNSCC was found to vary from 5.2 to 90% in previous studies (3, 5). This wide variation could be due to the small sample sizes and the heterogeneity of the patients. Thus, high-quality clinical datasets with large sample sizes are needed to analyze the survival, etiology and other clinical characteristics of PNI.

As PNI plays an important role in the progression of HNSCC, elucidating the mechanism of PNI is critical for facilitating the development of novel therapeutics that target PNI and can enhance patient survival and quality of life. However, the molecular changes that occur during clinical PNI in patients with HNSCC have not been well-investigated, and the mechanism of PNI is largely unknown. Only a handful of molecular studies have been performed to explore the roles of genes in the process of PNI (6–10). These studies have mainly focused on the functions of specific individual genes. However, advances in high-throughput technologies allow us to explore molecular changes at the omics level. Currently, there is no standard approach for analyzing transcriptomic data, and network medicine is a promising method for better understanding diseases by combining systems biology and network science to explore the network relationships of interacting components. This approach provides insights into these conditions beyond the level of a single gene. Weighted gene coexpression network analysis (WGCNA) (11) is a network method for identifying clusters or modules that are likely to be coregulated or work together in a biologically coherent fashion. A module can then be summarized as a single unit, which can be correlated with phenotypes, such as PNI.

The Cancer Genome Atlas (TCGA) has summarized the molecular characteristics of tumors at different omic levels, including the genomic, epigenomic, transcriptomic, and proteomic levels, and provided histopathological and clinical annotations with sufficient follow-up (12, 13). The resulting resource enabled us to analyze the relationships between PNI and clinical signatures and identify gene modules associated with PNI, leading to a comprehensive understanding of PNI from the clinical level to the molecular level. Due to advances in single-cell sequencing, it is possible to capture intratumoral heterogeneity among malignant and non-malignant cells; therefore, we also explored the PNI-associated gene modules in different cell types.

## Materials and Methods

### Data Collection

Gene expression data (500 cases) presented in fragments per kilobase million (FPKM) and the corresponding clinical information (528 cases) for HNSCC were downloaded from the official TCGA website on March 2017. Among the case data, data for 370 patients with an available PNI status were used to assess risk factors for PNI. Data for 319 patients with both an available PNI status and survival results were used to analyze the effects of PNI on prognosis. Data from 351 patients with gene expression data and PNI status data were used for WGCNA, gene set enrichment analysis (GSEA) and differential expression analysis.

### Prognostic Analysis

Survival analysis was conducted using the Kaplan-Meier method and log-rank tests. Hazard ratios (HRs) were calculated using a Cox proportional hazards model with R software and the “survminer” R package. In the survival analysis, death from any cause was considered an event. Forest plots were used to show survival differences between subgroups.

### Assessment of Risk Factors

We examined associations of age, gender, tobacco history, human papillomavirus (HPV), and other potential risk factors with the incidence of PNI using logistic regression models to estimate odds ratios (ORs) and 95% confidence intervals (CIs).

### WGCNA

The top 25% of the most variant genes, as determined by analysis of variance (6,249 genes), were selected for WGCNA. The WGCNA R package was used to construct a gene coexpression network. A soft-thresholding power of 5 was estimated and used to derive a pairwise distance matrix for the selected genes using the topological overlap measure (TOM), which quantified the degree of network neighbors. We used average linkage hierarchical clustering to classify genes with similar expression profiles into the same gene modules. The module eigengene (ME) was the first principal component of a module and summarized the characteristic expression pattern of that module. MEs were calculated to evaluate correlations between the modules and clinical traits (PNI status). Module membership (MM) is the Pearson correlation between the expression level of a given gene and a given ME. The top 12 genes with the highest MM in the significant module were identified as hub genes. For visual analysis of the constructed networks, the gene coexpression networks in the targeted module were represented using Cytoscape 3.6.1.

### Gene Ontology (GO) Biological Process Analysis

The Database for Annotation, Visualization and Integrated Discovery (DAVID, http://david.abcc.ncifcrf.gov/, version 6.8) was used to perform GO and Kyoto Encyclopedia of Genes and Genomes (KEGG) pathway analyses.

### Single-Cell Transcriptomic Analysis

The gene expression profiles in the GSE103322 dataset (https://www.ncbi.nlm.nih.gov/geo/query/acc.cgi?acc=GSE103322) submitted by Puram et al (14). were downloaded from the Gene Expression Omnibus (GEO) database. The single-cell dataset contained 5,902 single cells from 18 patients with oral cavity tumors. The distribution of the hub genes was explored among different cell types. A cancer single-cell database that collected 14 functional state signatures was also used to explore the functions of the genes in the targeted module. Then, gene set variation analysis (GSVA) and Spearman's rank correlation were used to compute the activities of the 14 functional states and the correlations between the activities and gene expression for 2,105 single cancer cells in GSE103322 (15).

### Differential Expression Analysis

The R package “limma” was used to screen the differentially expressed genes (DEGs) between the cases with PNI and without PNI. A volcano plot was constructed to show the DEGs.

### GSEA

GSEA was performed using GSEA 3.0 (http://www.broadinstitute.org/gsea/). A total of 351 HNSCC samples were divided into two groups according to the presence or absence of PNI. A nominal *p* < 0.05 and a false discovery rate (FDR) *q* < 0.25 were considered significant.

### Cox-Regression Analysis and Nomogram Construction

Univariate and multivariate analyses were performed using the Cox proportional hazards model. A nomogram plot was generated using the R package “rms.”

## Results

### PNI Significantly Affects Prognosis

**Figure 1:**
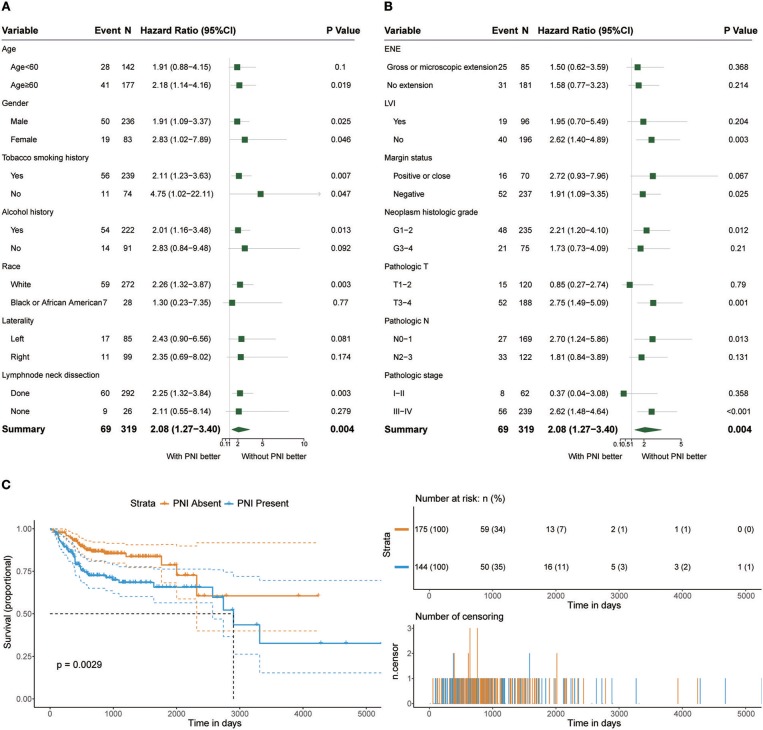
**(A,B)** Subgroup analysis of OS in patients with PNI and patients without PNI. **(C)** OS in patients with and without PNI.

**Figure 2:**
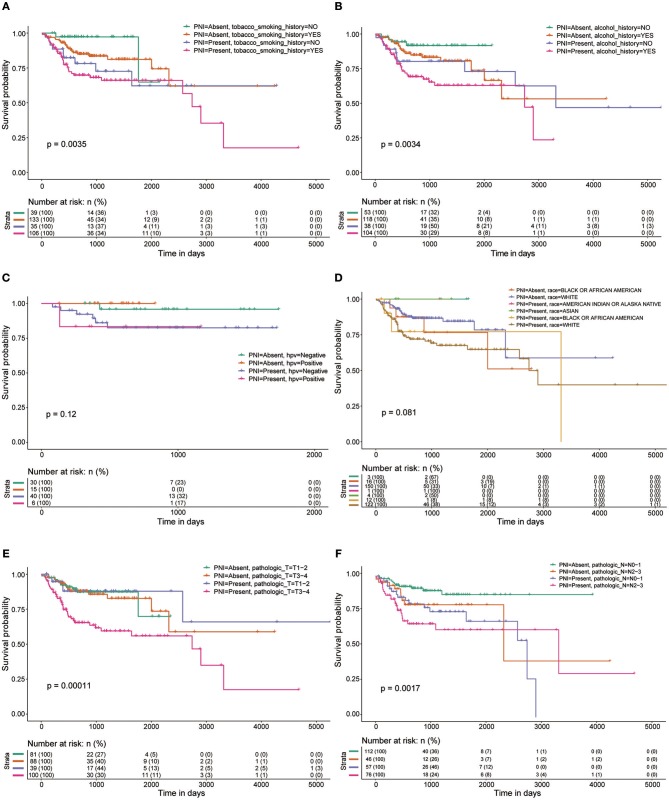
OS rates of patients with and without PNI were compared with regards to **(A)** smoking history, **(B)** alcohol history, **(C)** HPV status, **(D)** race, **(E)** pathologic T category, and **(F)** pathologic N category.

Among the 319 patients included in the survival analysis, we found that PNI was present in 144 patients (45.1%). Survival analyses showed that PNI was significantly inversely associated with overall survival (OS) (HR, 2.08; 95% CI, 1.27 to 3.40; *P* = 0.004; Figure 1). We performed an exploratory subgroup analysis of OS (Figures 1, 2) and found that PNI was significantly associated with worse OS in advanced-stage patients (pathologic stage III-IV) (HR, 2.62; 95% CI, 1.48 to 4.64; *P* < 0.001), while OS in early-stage patients was not influenced by PNI (HR, 0.37; 95% CI, 0.04 to 3.08; *P* = 0.358). For pathologic T1-2 categories, PNI was not significantly associated with OS (HR, 0.85; 95% CI, 0.27 to 2.74; *P* = 0.79). However, PNI decreased the OS of patients with T3-T4 lesions (HR, 2.75; 95% CI, 1.49 to 5.09; *P* = 0.001). For patients in N2-N3 categories, a trend toward worse OS with PNI was evident (HR, 1.81; 95% CI, 0.84 to 3.89; *P* = 0.131). The decreased survival of N0-1 patients with PNI was more significant (HR, 2.70; 95% CI, 1.24 to 5.86; *P* = 0.013). To compare PNI with other clinical variables with regard to the impact on prognosis, we also performed univariate (Supplementary Table 1) and multivariate (Supplementary Table 2) Cox regression analyses. In univariate analysis, age, extranodal extension(ENE), PNI, pathologic T category and pathologic N category were found to be prognostic factors. In addition, we constructed a nomogram to predict HNSCC prognosis using PNI and other clinical variables, however, the calibration and clinical use (decision curve analysis) of the nomogram remains to be validated using another cohort (Supplementary Figure 1).

### Assessment of Risk Factors

**Figure 3:**
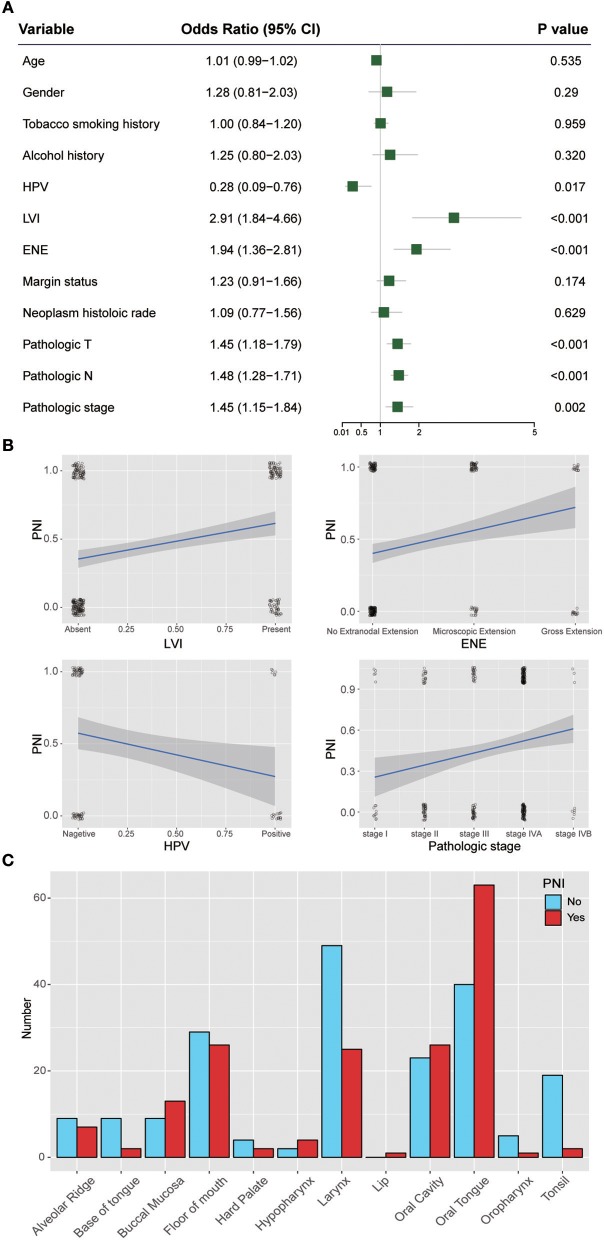
**(A)** Relative risk for PNI with regards to age, gender, smoking history, alcohol history, HPV status, LVI, ENE, margin status, neoplasm histologic grade, pathologic T category, pathologic N category, and pathologic stage. **(B)** Regression curve for the relationships of PNI with LVI, ENE, HPV status, and pathologic stage. **(C)** Relationship between PNI and the anatomical distribution of the primary tumor. HPV, human papillomavirus; LVI, lymphovascular invasion; ENE, extranodal extension.

Figure 3 shows the ORs of potential risk factors for PNI. We found that age, gender, smoking history, and alcohol history were not risk factors for PNI. Patients with lymphovascular invasion (LVI) (OR, 2.91; 95% CI, 1.84 to 4.66; *P* < 0.001) or ENE (OR, 1.94; 95% CI, 1.36 to 2.81; *P* < 0.001) were more likely than those without these factors to develop PNI. In addition, patients with a higher T category(OR, 1.45; 95% CI, 1.18 to 1.79; *P* < 0.001) or N category(OR, 1.48; 95% CI, 1.28 to 1.71; *P* < 0.001) were at risk of PNI. Notably, HPV-positive patients (OR, 0.28; 95% CI, 0.09 to 0.76; *P* = 0.017) were less likely than HPV-negative patients to develop PNI. We also analyzed the impact of anatomic neoplasm subdivision on the incidence of PNI. Oral tongue cancer appeared likely to develop PNI, while the incidence of PNI in other areas, such as the tonsils or the base of the tongue, was relatively low (Figure 3C).

### Weighted Coexpression Network Construction and Key Module Identification

**Figure 4:**
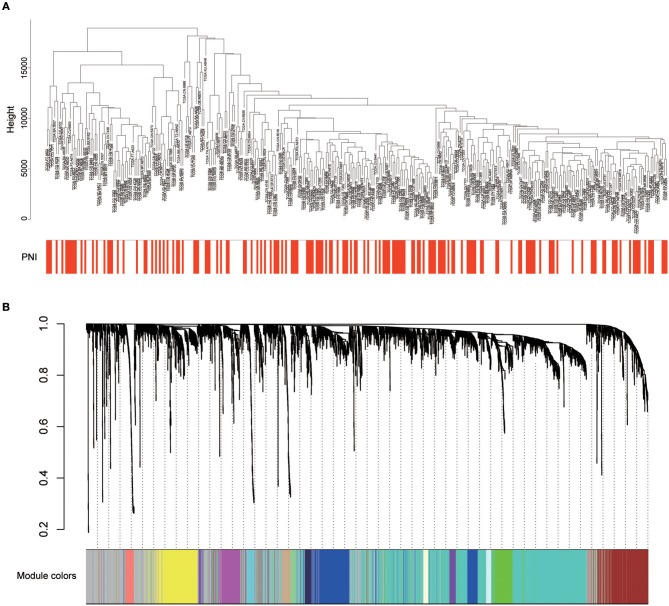
**(A)** Cluster analysis of HNSCC expression data after removing outliers. **(B)** Clustering dendrogram of genes. A hierarchical cluster analysis dendrogram was used to detect coexpression clusters. Each color is assigned to 1 module (gray represents unassigned genes).

**Figure 5:**
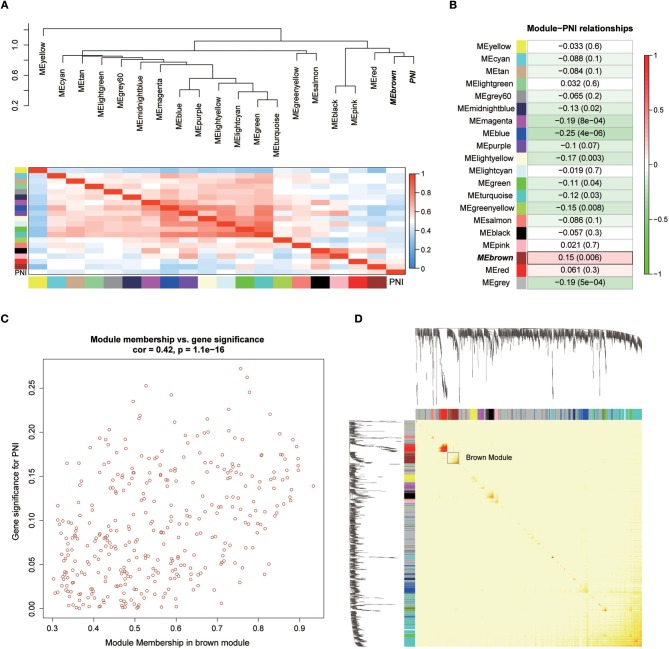
**(A)** Correlations between the modules. **(B)** Correlation values for different module-PNI relationships. **(C)** Scatter plot of the correlation between gene MM in the brown module, which was associated with PNI, and gene significance for PNI. **(D)** Network heatmap plot showing genes sorted into rows and columns by the clustering tree. Lighter colors denote lower adjacency, and darker colors denote higher adjacency.

**Figure 6:**
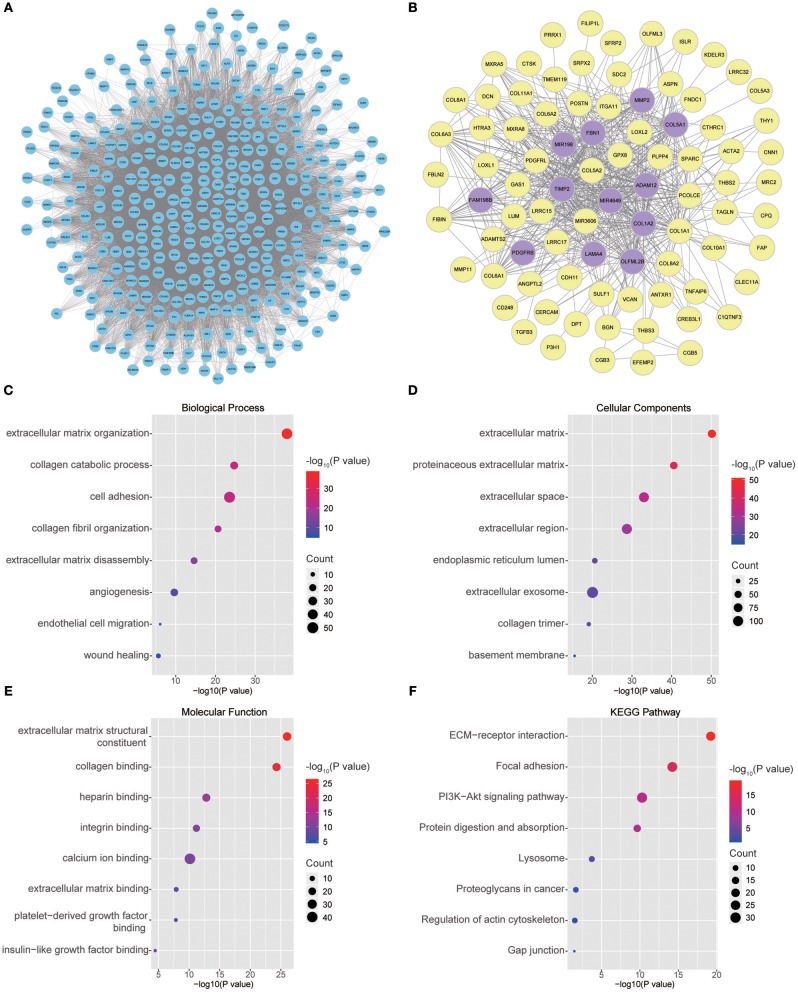
**(A)** Visual representation of the coexpression networks in the key module. **(B)** Coexpression networks when only a weight of more than 0.2, as calculated by WGCNA, was considered. The hub genes are highlighted. **(C–F)** GO and pathway enrichment analysis of brown module genes. **(C)** BP analysis. **(D)** CC analysis. **(E)** MF analysis. **(F)** KEGG pathway analysis.

Twenty-six outlier samples in TCGA were removed after clustering. The remaining 325 samples were used in subsequent analyses (Figure 4A). The 6,249 variant genes with the largest variance between samples were grouped into modules via average linkage hierarchical clustering. A value of β = 5 was selected as the soft-thresholding power to ensure a scale-free network. A total of 19 modules were identified (Figure 4B, Supplementary Data Sheet 2). Unassigned genes were categorized into gray modules. The relationships between PNI and the coexpression networks are presented in Figures 5A,B. The brown module was significantly associated with PNI (*P* = 0.006). The TOM was visualized with a heatmap that could depict adjacencies or topological overlaps (Figure 5D). The top 12 genes (TIMP2, MIR198, LAMA4, FAM198B, MIR4649, COL5A1, COL1A2, OLFML2B, MMP2, FBN1, ADAM12, and PDGFRB) with the highest MM in the brown module were identified as hub genes. The weighted coexpression network of the brown module is shown in Figures 6A,B (Supplementary Data Sheet 3).

### GO and Pathway Enrichment of the Key Module

To obtain further insight into the functions of the genes in the brown module, the gene list was uploaded to DAVID. GO analysis involving three categories, biological process (BP), molecular function (MF), and cellular component (CC), and KEGG pathway analysis were carried out. The GO analysis results showed that genes were mainly enriched in extracellular matrix (ECM) organization, collagen catabolic processes, cell adhesion, collagen fibril organization, ECM disassembly, angiogenesis, endothelial cell migration and wound healing in the BP category (Figure 6C). The genes in the brown module were enriched in the ECM region in the CC category (Figure 6D). For the MF category, the genes were enriched in ECM structural constituents, collagen binding, integrin binding, calcium ion binding, ECM binding, platelet-derived growth factor binding and insulin-like growth factor binding (Figure 6E). The results of KEGG pathway analysis showed that the genes were mainly involved in ECM-receptor interactions, focal adhesion, the PI3K-Akt signaling pathway, protein digestion and absorption, lysosomes, proteoglycans in cancer, regulation of the actin cytoskeleton and gap junctions (Figure 6F).

### Functional State Signatures of Single Cancer Cells

**Figure 7:**
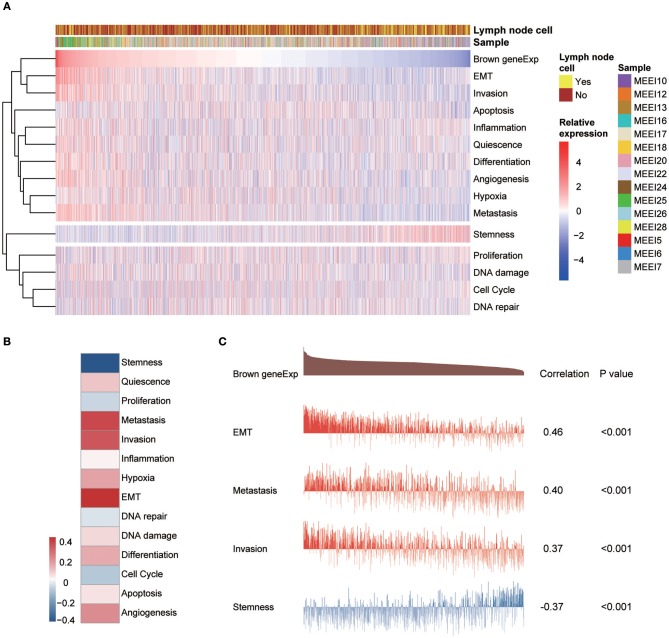
**(A)** Heatmap showing the correlations between the expression of key module genes and functional state signatures from an analysis of 2,105 single cancer cells in the GSE103322 dataset. MEE5, MEE6, MEE7, MEE10, MEE12, MEE13, MEE16, MEE17, MEE18, MEE20, MEE22, MEE24, MEE25, MEE26, and MEE28 represent cells from different patients. The sources of the cells (primary lesion or metastatic lymph node) are noted. **(B)** Heatmap of the correlations between gene expression and 14 functional states. **(C)** Details of the relationships between brown module gene expression and “EMT,” “metastasis,” “invasion,” and “stemness”.

A study by the Broad Institute (14) represents the largest effort thus far to evaluate single-cell transcriptomic expression profiles of primary and metastatic HNSCC. The data were used in this study to explore the functional states of the genes in the module associated with PNI in single HNSCC cells. Among the 14 functional state signatures collected by the CancerSEA database (15), the expression of the brown module genes in 2,105 cancer cells was found to be positively correlated with the signatures “EMT” (epithelial-mesenchymal transition) (correlation, 0.46; *P* < 0.001), “metastasis” (correlation, 0.40; *P* < 0.001) and “invasion” (correlation, 0.37; *P* < 0.001). We also found a negative correlation between the expression of key module genes and “stemness” (correlation, −0.37; *P* < 0.001) (Figures 7A and 7C). The correlations with other functional state signatures, including “angiogenesis” (correlation, 0.25; *P* < 0.001), “hypoxia” (correlation, 0.20; *P* < 0.001), “differentiation” (correlation, 0.20; *P* < 0.001), “quiescence” (correlation, 0.13; *P* < 0.001), “DNA damage” (correlation, 0.10; *P* < 0.001), “apoptosis” (correlation, 0.08; *P* < 0.001), “inflammation” (correlation, 0.06; *P* < 0.01) and “cell cycle” (correlation, −0.06; *P* < 0.01), are shown in Figure 7B.

### Key Module Gene Distribution in Different Cell Types

**Figure 8:**
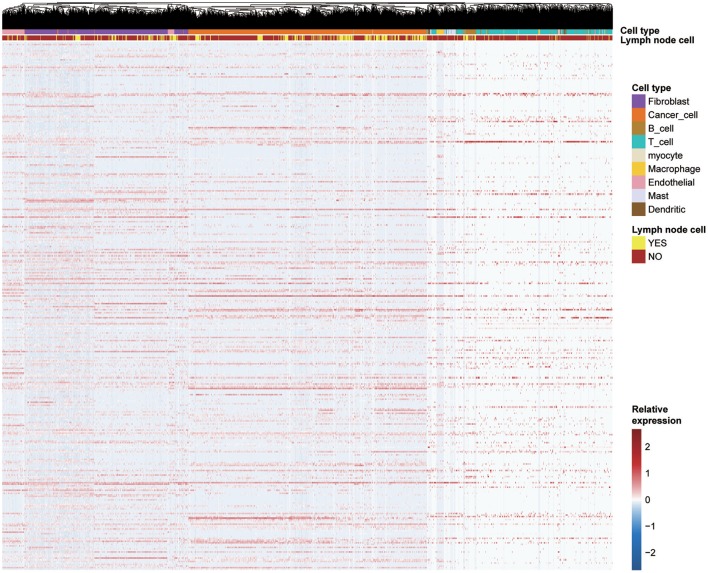
Expression levels of all genes (357 genes) in the key coexpression module among 5,902 malignant and non-malignant single cells.

**Figure 9:**
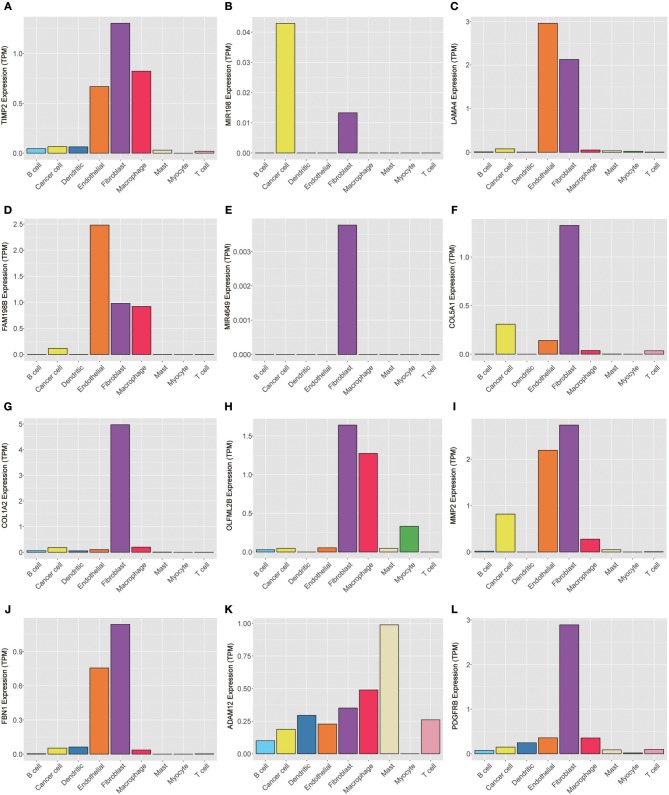
Expression levels of the hub genes **(A)** TIMP2, **(B)** MIR198, **(C)** LAMA4, **(D)** FAM198B, **(E)** MIR4649, **(F)** COL5A1, **(G)** COL1A2, **(H)** OLFML2B, **(I)** MMP2, **(J)** FBN1, **(K)** ADAM12, and **(L)** PDGFRB among 5,902 malignant and non-malignant single cells.

To explore the genes in the brown module, which was significantly associated with PNI, we analyzed the distribution of the genes in the GSE103322 dataset, which contains the expression profiles of 5,902 single cells. The results are shown in the form of a heatmap (Figure 8). The distribution of hub genes (TIMP2, MIR198, LAMA4, FAM198B, MIR4649, COL5A1, COL1A2, OLFML2B, MMP2, FBN1, ADAM12, and PDGFRB) was further evaluated. Among the different cell types, the expression of most of the hub genes (TIMP2, MIR4649, COL5A1, COL1A2, OLFML2B, MMP2, FBN1, and PDGFRB) was highest in fibroblasts. The expression of MIR198, LAMA4, FAM198B, and ADAM12 was also relatively high in fibroblasts. In addition, the expression levels of the hub genes were relatively high in endothelial cells and macrophages (Figure 9).

### GSEA

We conducted GSEA between cases with and without PNI in TCGA. GSEA revealed significant differences (FDR < 0.05, nominal *p* < 0.05) in the enrichment of “HALLMARK_EMT,” “HALLMARK_ANGIOGENESIS,” “HALLMARK_COAGULATION,” “KEGG_ECM_RECEPTOR,” “KEGG_FOCAL_ADHESIN,” “GO_ACTIN_FILAMENT_BUNDLE,” “GO_ACTOMYOSIN,” “GO_CELL_SUBSTRATE_JUNCTION,” and “GO_LAMELLIPODIUM_MEMBRANE” (c2.cp.kegg.v6.2.symbols, h.all.v6.2, c5.bp.v6.0.symbols, c5.cc.v6.0.symbols, and c5.mf.v6.0.symbols) (Discrepancy: Supplementary Figure 2).

### Differential Expression Analysis

Under the threshold of FDR < 0.05 and |log 2-fold change|>1, a total of 161 DEGs (92 upregulated and 69 downregulated in PNI samples in TCGA) were identified (Supplementary Data Sheet 1; Supplementary Figure 3).

## Discussion

Solid tumors, such as those in patients with head and neck cancer, pancreatic cancer(16), colorectal cancer(17), and prostate cancer(18), disseminate primarily via three processes: direct invasion of the surrounding tissue, lymphatic spread, and hematogenous spread. However, a fourth process, dissemination along nerves (19), is frequently ignored and remains poorly understood. PNI, a histologic finding of tumor cell infiltration, differs from perineural tumor spread, the behavior by which a macroscopic tumor spread along a nerve (20). This study aimed to perform a thorough analysis of PNI in HNSCC from the clinical level to the molecular level and presents the first description of cancer-related PNI from the omics perspective to date.

We found that PNI in HNSCC is correlated with poor clinical outcomes, which is consistent with the results of previous studies (21–24). Subgroup analysis, which is important for determining populations most in need of intervention measures, was performed in the current study. Adding insult to injury, PNI was shown to be significantly associated with poor OS in patients at advanced stages. However, the survival of early-stage patients was not markedly influenced by PNI. These results indicate that more attention should be paid to PNI in advanced patients, and more clinical studies on treatments for such patients are needed.

In a recent study of 178 patients with HNSCC, tumors of current and former smokers showed PNI significantly more often than did tumors of patients who had never smoked (25). However, we found that tobacco smoking history was not a risk factor for PNI in HNSCC. In addition, age, gender, and alcohol history were not significant factors associated with the presence of PNI (*P* > 0.05). Notably, HPV-positive patients are less likely than HPV-negative patients to develop PNI. These results were inconsistent with those of a previous study that included 71 patients and found that HPV had no impact on PNI (26). However, only 97 patients whose HPV status was identified by *in situ* hybridization (ISH) or p16 positivity in the HNSCC project in TCGA were used to assess the impact of HPV on PNI; thus, high-quality studies with larger sample sizes are needed to verify our findings. We also found that tumors from different anatomic sites had different risks of PNI, and oral tongue cancer was associated with a relatively high risk of PNI. These differences could be caused by differences in innervation in different areas. One limitation of this study is that the depth of invasion of the primary tumor, a potential risk factor for PNI, was unavailable in TCGA. Another limitation that should be noted is that there were several grades of PNI in the careful examination of histological specimen, which were indistinguishable in the TCGA cohort. More details about PNI should be included in further studies on PNI in HNSCC or other cancers.

Treatment considerations of PNI are hampered by the lack of higher levels of evidence, such as randomized clinical trials, to form conclusions or reliable guidelines. For patients with detected PNI with its extent defined with preoperative imaging methods such as magnetic resonance neurography, surgical management will enable us to obtain better tumor control (27, 28). However, in the clinic, PNI was always found during pathologic specimen examination after resection of the primary tumor, and appropriate radiation therapy, given as definitive, adjuvant or salvage treatment, can improve local control in patients with PNI. The authors of a recent review recommended adjuvant radiation in the setting of head and neck mucosal squamous cell carcinoma with extensive microscopic PNI (20). However, whether patients with “non-extensive” PNI could benefit from radiation needs further study due to the lack of reliable evidence and clinical decisions should be made with caution. Novel therapeutic strategies, such as targeted drug administration, are expected with the further growth of understanding of the molecular basis for PNI.

The molecular mechanisms of PNI are still largely unknown, and targeted treatment for PNI is lacking. With the development of sequencing techniques, studies have transitioned from investigating individual genes to systematically integrating omics information from cancer tissues. TCGA has illuminated the transcriptomic landscape of HNSCC, providing an opportunity to use network tools such as WGCNA to integrate omics data for a better understanding of the molecular changes that occur during the process of PNI. We identified a coexpression network module, the only module significantly associated with PNI, that consisted of 357 genes, including matrix metalloproteinases, laminins, and integrins. GO enrichment analysis indicated that the genes in the key module function mainly in the BPs of ECM organization, collagen catabolic processes and cell adhesion, which could reflect the course of invasion. Pathway enrichment analysis indicated that the genes in the key module were enriched in the PI3K-Akt signaling pathway, which is likely to play an important role in PNI in HNSCC. A recent study found that HNSCC with histological PNI had increased levels of Akt/PKB and mTOR kinase activation (29), and the Akt pathway has also been found to play a role in PNI in pancreatic cancer (30).

It is essential to detect PNI, as patients with PNI may require more aggressive treatment (27, 31). Despite the capacity of magnetic resonance imaging (MRI) and F-fluorodeoxyglucose positron emission tomography (F-FDG PET) to assess PNI in HNSCC, early-stage PNI is rarely detected (32, 33). The hub genes in the key module associated with PNI and the DEGs found in differential expression analysis in our study could be potential biomarkers of PNI; however, the efficacy of these potential biomarkers requires further confirmation through immunohistochemical (IHC) studies.

TCGA provides an atlas of high-quality transcriptome data. However, these data rely on sequencing technologies that measure tumors in bulk, limiting the ability to capture intratumoral heterogeneity among different cells. Intratumoral heterogeneity among malignant and non-malignant cells and interactions within the tumor microenvironment (TME) have been found to be critical to tumorigenesis (34). Recently, scientists have profiled the transcriptomes of ~6,000 single cells from 18 HNSCC patients (14). We analyzed the functions of key module genes in this independent dataset and found strong correlations between gene expression and the functional states “EMT,” “metastasis,” and “invasion.” Similarly, GSEA indicated a strong correlation between the mRNA levels of the key module genes and the predefined EMT gene signatures. Initially, the predominant theory of PNI was that “tumor cells spread passively along planes of least resistance in the connective tissues cover[ing] the nerve, thus presenting PNI as a type of simple diffusion.” However, ultrastructural studies have shown that “multiple layers of collagen and basement membrane compose the nerve sheath and make this path highly resistant” (5, 35). Thus, PNI was deemed a process of invasion rather than simple diffusion. However, this theory remained unverified at the molecular level, constrained by the sequencing technology of past decades. The findings obtained in our study provided direct evidence that PNI is an invasive process. Interestingly, EMT programs and stemness are always intimately linked in cancer cells; EMT programs favor the acquisition of carcinoma cell stemness, yielding tumor-initiating cells. However, our findings indicated that the key coexpression module was highly correlated with EMT and negatively associated with stemness. The stemness associated with EMT could be due to EMT-activating transcription factor (EMT-TF)-dependent dedifferentiation and plasticity (36–38). The authors of the single-cell transcriptomic analysis used in our study found that EMT in HNSCC was more of a partial EMT (p-EMT) program than a complete EMT program, with ECM proteins that lacked classic EMT transcription factors (TFs). In this p-EMT program, expression of the classic EMT TFs ZEB1/2, TWIST1/2, and SNAIL1 was not detected. Only SNAIL2 was detected in 70% of HNSCC cells (14). Thus, p-EMT is a possible explanation for these conflicting results. IHC analyses (14) have also shown that p-EMT cells localize to the leading edge of primary tumors, potentially enabling the cancer cells to invade the peri- and intra-neural areas.

To perform a preliminary analysis of the role of non-malignant cells in PNI, we evaluated the expression levels of the genes in the key coexpression module, especially the hub genes. We found that these genes were mainly enriched in fibroblasts, an important component of the tumor stroma that contributes to the shaping of the ECM, the metabolic and immune reprogramming of the TME, and the release of growth factors that promote tumor growth and invasion (39, 40). Interestingly, cancer-associated fibroblasts (CAFs) have been found to be in close proximity to p-EMT cells in the surrounding TME. It has also been found that “tumors with both high CAF scores and high p-EMT scores have a particularly high propensity for invasion and metastasis, consistent with a cooperative effect” (14). All things considered, CAFs probably play an important role in PNI, which is a type of invasion. Finally, it is important to address the limitation that Schwann cells and stellate cells, which have been reported to be indispensable for PNI (5, 41, 42), were not identified in this single-cell sequencing study, possibly because of the lack of reliable biomarkers.

## Conclusion

In summary, our work provides important insights into PNI in HNSCC. PNI was found to be associated with poor survival, especially in patients with advanced HNSCC. In addition, HPV-negative HNSCC cases were found to be more likely than HPV-positive cases to develop PNI. By analyzing data from TCGA, we identified a PNI-associated coexpression module that consists of genes that function in processes such as ECM remodeling, collagen catabolic processes and cell adhesion. In addition, in analyses of single-cell transcriptomic data, we found that the expression of the genes in the PNI-associated module in cancer cells was correlated with the functional states “EMT,” “metastasis,” and “invasion.” CAFs probably play an important role in PNI given their relatively high expression levels of the key genes. Finally, we verified that PNI in HNSCC is a process of invasion rather than simple diffusion.

## Author Contributions

ZZ, JL, and XW conceived and designed the experiments. ZZ, RJ, YS, and XL collected data. ZZ and RL performed the statistical analysis. ZZ, TL, YF, and XW supported the experiments and helped to draft the manuscript. RL, XW, and JL wrote the manuscript. All authors read and approved the final manuscript.

### Conflict of Interest Statement

The authors declare that the research was conducted in the absence of any commercial or financial relationships that could be construed as a potential conflict of interest.

## Supplementary Material

Supplementary Figure 1 Nomogram indicating the survival probability of patients with HNSCC. The subscore is determined by drawing a line straight up to the point axis to establish the score associated with the variables of age, gender, ENE, PNI, pathologic T category, pathologic N category. The total score is obtained by adding subscores. A line is drawn straight down to the survival probability axis of 1, 3, and 5 years to obtain the probability. ENE, extranodal extension.

Supplementary Figure 2 Enrichment plots from GSEA showing that the gene sets for **(A)** EMT, **(B)** angiogenesis, **(C)** coagulation, **(D)** ECM-receptor interactions, **(E)** focal adhesion, **(F)** actin filament bundles, **(G)** actomyosin, **(H)** cell substrate junctions, and **(I)** the lamellipodium are differentially enriched in patients with PNI in TCGA. **(J)** The expression of genes in the EMT gene set in cases with and without PNI is shown. NES, normalized enrichment score.

Supplementary Figure 3 Volcano plot depicting gene expression differences in tumors with PNI vs. tumors without PNI in TCGA.

Supplementary Table 1 Univariate Cox regression analysis of various parameters in the TCGA cohort.

Supplementary Table 2 Multivariate Cox regression analysis of various parameters in the TCGA cohort.

Supplementary Data Sheet 1 DEGs identified by the “limma” R package between patients with PNI and patients without PNI in TCGA.

Supplementary Data Sheet 2 Details of the results of WGCNA.

Supplementary Data Sheet 3 Relationships among genes in the brown module. The coexpression network was visualized in Cytoscape.
