# Supplementary figures and images for: Integrating Clinical and Genetic Analysis of Perineural Invasion in Head and Neck Squamous Cell Carcinoma
Source: Front Oncol. 2019 May 31;9:434. doi: 10.3389/fonc.2019.00434 (PMC6555133; doi:10.3389/fonc.2019.00434)

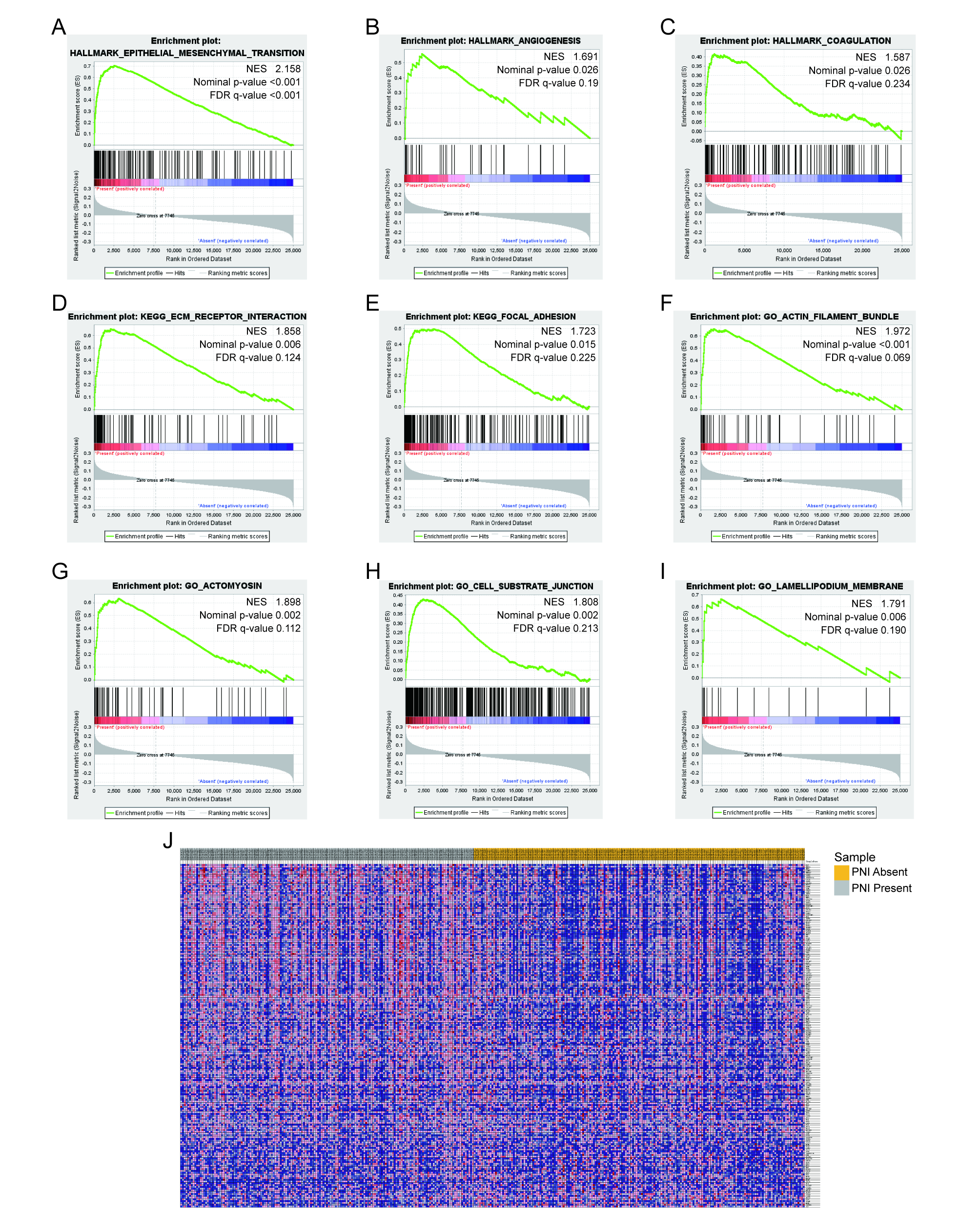

Supplement: Supplementary Figure 1 — Nomogram indicating the survival probability of patients with HNSCC. The subscore is determined by drawing a line straight up to the point axis to establish the score associated with the variables of age, gender, ENE, PNI, pathologic T category, pathologic N category. The total score is obtained by adding subscores. A line is drawn straight down to the survival probability axis of 1, 3, and 5 years to obtain the probability. ENE, extranodal extension. [file Image_1.TIF]

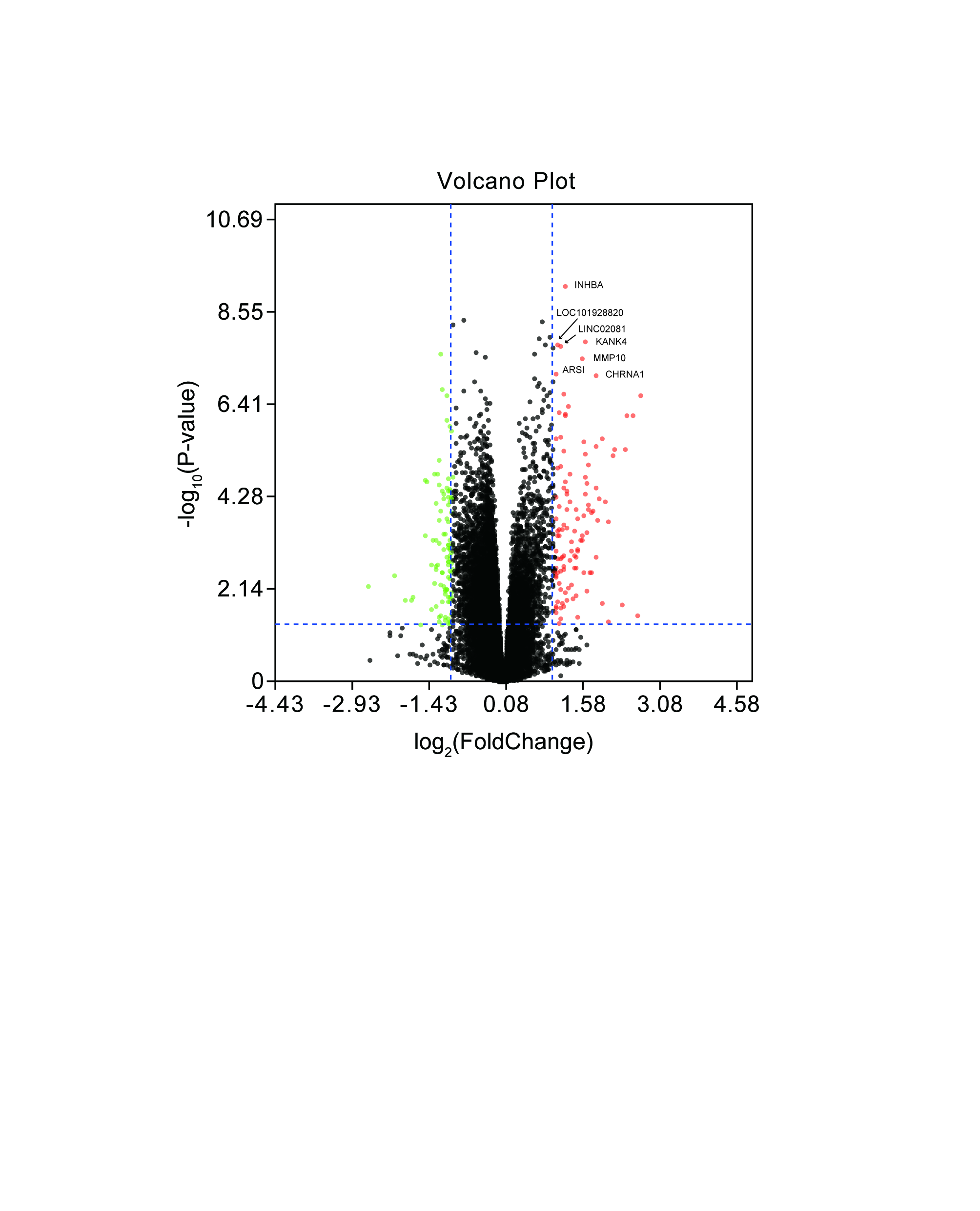

Supplement: Supplementary Figure 2 — Enrichment plots from GSEA showing that the gene sets for (A) EMT, (B) angiogenesis, (C) coagulation, (D) ECM-receptor interactions, (E) focal adhesion, (F) actin filament bundles, (G) actomyosin, (H) cell substrate junctions, and (I) the lamellipodium are differentially enriched in patients with PNI in TCGA. (J) The expression of genes in the EMT gene set in cases with and without PNI is shown. NES, normalized enrichment score. [file Image_2.TIF]

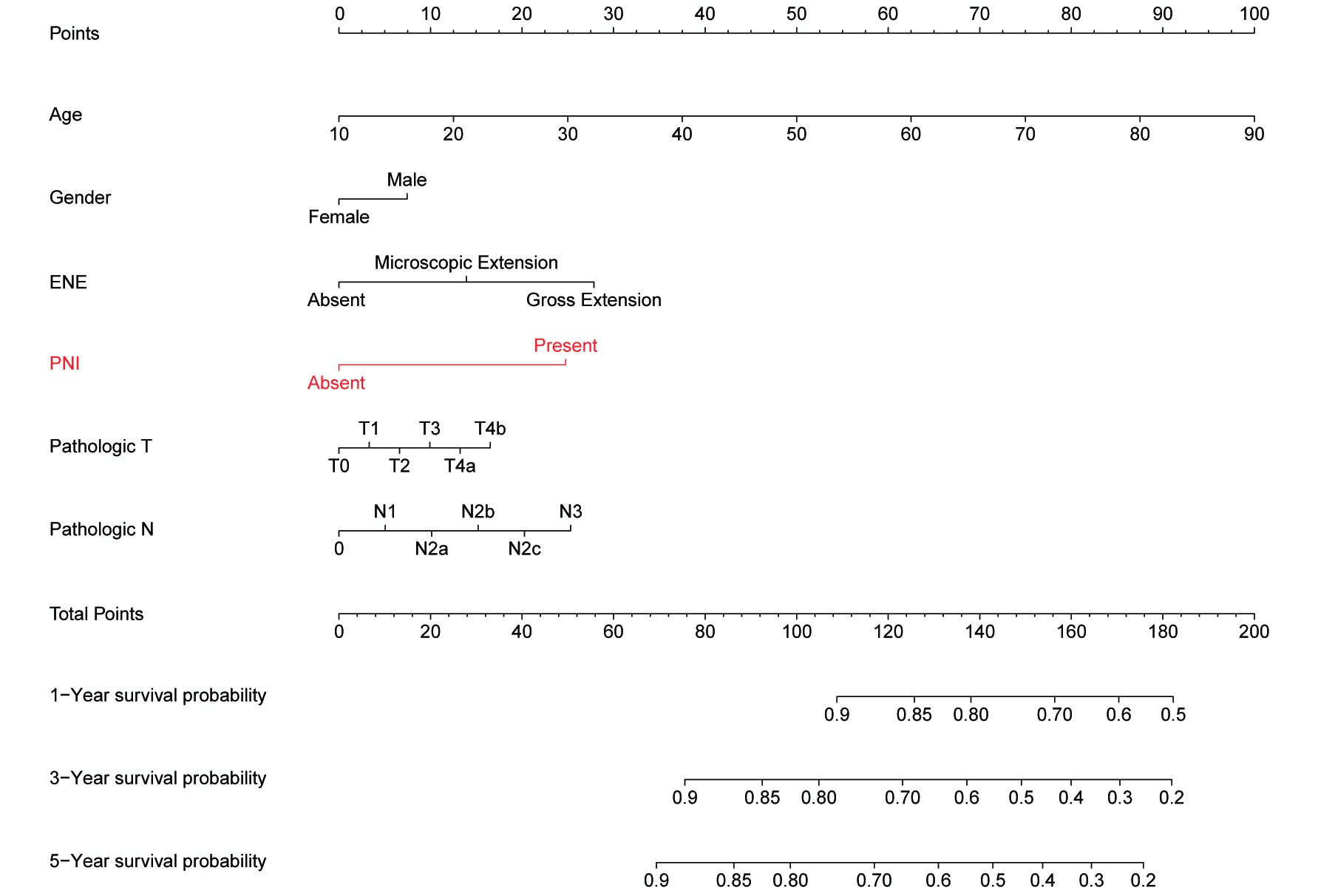

Supplement: Supplementary Figure 3 — Volcano plot depicting gene expression differences in tumors with PNI vs. tumors without PNI in TCGA. [file Image_3.TIF]
