# Supplementary material for: Integrating Clinical and Genetic Analysis of Perineural Invasion in Head and Neck Squamous Cell Carcinoma
Source: Front Oncol. 2019 May 31;9:434. doi: 10.3389/fonc.2019.00434 (PMC6555133; doi:10.3389/fonc.2019.00434)
Supplement: Supplementary Table 1 — Univariate Cox regression analysis of various parameters in the TCGA cohort. [file Table_1.DOCX]

| **Supplementary Table 1** Univariate cox-regression analysis of various parameters | | | |
| --- | --- | --- | --- |
| Clinicopathologic variable | Hazard Ratio | 95% confidence interval | P value |
| Gender | 1.442 | 0.974-2.135 | 0.068 |
| Age | 1.031 | 1.014-1.049 | <0.001 |
| Alcohol history | 1.035 | 0.692-1.550 | 0.865 |
| Tobacco smoking history | 1.105 | 0.935-1.306 | 0.241 |
| HPV | 0.263 | 0.032-2.136 | 0.211 |
| Margin status | 1.114 | 0.846-1.467 | 0.444 |
| LVI | 1.091 | 0.647-1.842 | 0.744 |
| ENE | 1.795 | 1.332-2.418 | <0.001 |
| **PNI** | **2.081** | **1.272-3.403** | **0.004** |
| Histologic grade | 1.081 | 0.826-1.414 | 0.572 |
| Pathologic T^*^ | 1.333 | 1.095-1.623 | 0.004 |
| Pathologic N^*^ | 1.224 | 1.073-1.397 | 0.003 |
| Anatomical distribution (Reference to oral tongue) | |  |  |
| Tonsil | 0.467 | 0.181-1.217 | 0.120 |
| Floor of mouth | 1.256 | 0.667-2.363 | 0.480 |
| Larynx | 0.809 | 0.470-1.394 | 0.445 |
| Alveolar ridge | 0.674 | 0.204-2.224 | 0.517 |
| Base of tongue | 0.448 | 0.136-1.483 | 0.189 |
| Oral cavity | 1.466 | 0.847-2.536 | 0.172 |
| Buccal mucosa | 0.850 | 0.327-2.209 | 0.739 |
| Oropharynx | 0.850 | 0.327-2.210 | 0.739 |
| Hard palate | 0.463 | 0.063-3.422 | 0.451 |
| Hypopharynx | 1.188 | 0.282-5.003 | 0.815 |
| Lip | 1.179 | 0.160-8.696 | 0.872 |
| ^HPV, human papillomavirus; LVI, lymphovascular invasion; ENE, extranodal extension; PNI, perineural invasion. *specific^ ^categories treated as different categorical variable(such as T4a and T4b, N2a, N2b and N2c)^ | | | |
