# Supplementary material for: Integrating Clinical and Genetic Analysis of Perineural Invasion in Head and Neck Squamous Cell Carcinoma
Source: Front Oncol. 2019 May 31;9:434. doi: 10.3389/fonc.2019.00434 (PMC6555133; doi:10.3389/fonc.2019.00434)
Supplement: Supplementary Table 2 — Multivariate Cox regression analysis of various parameters in the TCGA cohort. [file Table_2.DOCX]

| **Supplementary Table 2** Multivariate cox-regression analysis of various parameters | | | |
| --- | --- | --- | --- |
| Clinicopathologic variable | Hazard Ratio | 95% confidence interval | P value |
| Age | 1.030 | 1.002-1.059 | 0.036 |
| ENE | 1.435 | 0.915-2.251 | 0.115 |
| **PNI** | **1.833** | **0.990-3.396** | **0.054** |
| Pathologic T^*^ | 1.091 | 0.789-1.509 | 0.598 |
| Pathologic N^*^ | 1.139 | 0.908-1.428 | 0.262 |
| ^ENE, extranodal extension; PNI, perineural invasion. *specific^ ^categories treated as different categorical variable(such as T4a and T4b; N2a, N2b and N2c)^ | | | |
